# Bayesian and deep‐learning models applied to the early detection of ovarian cancer using multiple longitudinal biomarkers

**DOI:** 10.1002/cam4.7163

**Published:** 2024-04-10

**Authors:** Luis Abrego, Alexey Zaikin, Ines P. Marino, Mikhail I. Krivonosov, Ian Jacobs, Usha Menon, Aleksandra Gentry‐Maharaj, Oleg Blyuss

**Affiliations:** ^1^ Department of Women's Cancer EGA Institute for Women's Health, University College London London UK; ^2^ Department of Mathematics University College London London UK; ^3^ Department of Biology and Geology, Physics and Inorganic Chemistry Universidad Rey Juan Carlos Madrid Spain; ^4^ Research Center for Trusted Artificial Intelligence Ivannikov Institute for System Programming of the Russian Academy of Sciences Moscow Russia; ^5^ Institute of Biogerontology Lobachevsky State University Nizhny Novgorod Russia; ^6^ MRC Clinical Trials Unit University College London London UK; ^7^ Wolfson Institute of Population Health Queen Mary University of London London UK; ^8^ Department of Pediatrics and Pediatric Infectious Diseases, Institute of Child's Health Sechenov First Moscow State Medical University (Sechenov University) Moscow Russia

**Keywords:** CA125, change‐point detection, longitudinal biomarkers, ovarian cancer, recurrent neural networks

## Abstract

**Background:**

Ovarian cancer is the most lethal of all gynecological cancers. Cancer Antigen 125 (CA125) is the best‐performing ovarian cancer biomarker which however is still not effective as a screening test in the general population. Recent literature reports additional biomarkers with the potential to improve on CA125 for early detection when using longitudinal multimarker models.

**Methods:**

Our data comprised 180 controls and 44 cases with serum samples sourced from the multimodal arm of UK Collaborative Trial of Ovarian Cancer Screening (UKCTOCS). Our models were based on Bayesian change‐point detection and recurrent neural networks.

**Results:**

We obtained a significantly higher performance for CA125–HE4 model using both methodologies (AUC 0.971, sensitivity 96.7% and AUC 0.987, sensitivity 96.7%) with respect to CA125 (AUC 0.949, sensitivity 90.8% and AUC 0.953, sensitivity 92.1%) for Bayesian change‐point model (BCP) and recurrent neural networks (RNN) approaches, respectively. One year before diagnosis, the CA125–HE4 model also ranked as the best, whereas at 2 years before diagnosis no multimarker model outperformed CA125.

**Conclusions:**

Our study identified and tested different combination of biomarkers using longitudinal multivariable models that outperformed CA125 alone. We showed the potential of multivariable models and candidate biomarkers to increase the detection rate of ovarian cancer.

## Graphical abstract

Using the data from largest prospective ovarian cancer clinical trial, we assessed, for the first time, the performance of deep and statistical learning approaches in evaluating of the panel longitudinal biomarkers. Our results demonstrate that these models outperform CA125, the single best ovarian cancer biomarker. These findings underscore the potential of multimarker models in improving the detection rate of ovarian cancer and have significant implications for the field of cancer screening and early detection.

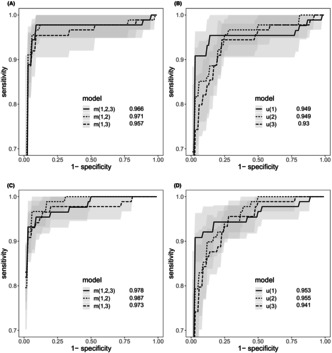

## 1 INTRODUCTION

Ovarian cancer is the most lethal of all gynecological cancers. When detected at early stage, the survival is much more encouraging (5‐year survival of >93% for Stage I disease) than when diagnosed at an advanced stage (5‐year survival of 13% for Stage IV).^1^ Despite the extensive efforts to improve treatment over the last 20 years, although there have been modest improvements in survival, these have not had a significant impact. The major efforts in detecting ovarian and tubal cancer have spanned decades. Screening trials using Cancer Antigen 125 (CA125) interpreted using a cutoff have not shown any mortality benefit.^2^, ^3^, ^4^ Algorithm‐based approaches to screening in the UK trial have demonstrated that longitudinal CA125 can lead to earlier detection (stage shift with multimodal screening) with no impact on mortality from the disease.^5^ More recent data however suggest that there may be longer survival in women diagnosed with the most lethal subtype of ovarian cancer, the high‐grade serous cancers (HGSC) in the screened (multimodal group) compared to the control group. Since its identification in 1981, CA125 has been used in clinical practice and investigated in screening trials. Clinical decisions have been made based on the patients' risk of having a change point in the serial CA125 with respect to their baseline. A statistical method to determine in a probabilistic way such a risk was developed (Risk of Ovarian Cancer Algorithm, ROCA).^6^, ^7^ It has not been until more recently (despite many international efforts) that HE4 had been identified as the second‐best marker in screening after CA125 (73% vs. 86% for CA125).^8^, ^9^, ^10^, ^11^, ^12^ The efforts have since focused on exploring the value of a combination of CA125, HE4, and other promising markers in combination. Furthermore, p53 autoantibodies have been shown to detect ovarian/tubal cancers in women with ovarian /tubal cancers which do not express CA125 (16% and many months prior to diagnosis, lead time of 22 months).^13^ The interpretation of multiple markers in longitudinal samples is challenging unless sophisticated mathematical modeling is applied. We have previously shown that a method of mean trends (MMT) algorithm has a comparable performance to the ROCA which was used in the UK trial (UK Collaborative Trial of Ovarian Cancer, UKCTOCS).^14^ Here, we extend the observation made in^14^, ^15^, ^16^ and describe a novel approach to interpretation of multiple markers in samples preceding diagnosis to assess if any of these can improve on sensitivity of the ROCA or offer potential advantage on lead time to detection of ovarian/tubal cancer.

## 2 METHODS

### 2.1 Change‐point detection algorithm

#### 2.1.1 Joint multivariable fully Bayesian model

Biomarker levels $Y_{\mathrm{ijk}}$ are modeled using a hierarchical Bayesian model (Figure S1). Here, subject‐specific variables are indexed by $i=1,2,\ldots ,n_{0},n_{0}+1,\ldots ,N$, where $\;n_{0}$ is the number of controls and the remaining subjects account as cases. A specific biomarker is indexed by k=1,2,…,K. Each patient i has a set of screening visits $\;t_{\mathrm{ij}}$ from zero up to time of last measurement $d_{i}$ (in years), where $j=1,2,\ldots ,T_{i}$.

Following the assumptions introduced in^7^ to model the cancer progression based on fully Bayesian screening, the longitudinal observations of biomarkers vary based on the nature of the patient. For control patients, the biomarker levels are expected to randomly fluctuate around a constant mean $\theta_{\mathrm{ik}}$. That is expressed as $Y_{\mathrm{ijk}}=\theta_{\mathrm{ik}}+\epsilon_{\mathrm{ijk}}$, with $\epsilon_{\mathrm{ijk}}∼N\left(0,\sigma_{k}^{2}\right)$. For cases, we define a binary indicator $I_{\mathrm{ik}}$ to distinguish between two different model assumptions in the evolution of biomarkers. If $I_{\mathrm{ik}}=0$, then we assume that the marker level does not increase after the onset of cancer and follows the same behavior modeled for controls. If $I_{\mathrm{ik}}=1$, the marker levels vary around a mean $\theta_{\mathrm{ik}}$ until an unobserved change‐point time, defined by $\tau_{\mathrm{ik}}$. From this change point, we expect a positive slope $\gamma_{\mathrm{ik}}$ of the biomarker levels up to diagnosis. That is $Y_{\mathrm{ijk}}=\theta_{\mathrm{ik}}+\gamma_{\mathrm{ik}}{\left(t_{\mathrm{ij}}-\tau_{\mathrm{ik}}\right)}^{+}+\epsilon_{\mathrm{ijk}}$ where ${\left(.\right)}^{+}$ is the positive part of the expression.

Let $S=\left\{\theta_{\mathrm{ik}},I_{i},\log\left(\gamma_{\mathrm{ik}}\right),\tau_{\mathrm{ik}}\right\}$ be the set of subject‐specific parameters. Thus, the probability density function of the observations conditional on the set of parameters S is

(1) $$\;P\left(Y|S;t\right)=\prod_{i=1}^{n_{0}}\prod_{k=1}^{K}\prod_{j=1}^{T_{i}}\varphi \left(\frac{\;Y_{\mathrm{ijk}}-\theta_{\mathrm{ik}}}{\;\sigma_{k}}\right)\times \prod_{i=n_{0}+1}^{N}\prod_{k=1}^{K}\prod_{j=1}^{T_{i}}\varphi {\left(\frac{\;Y_{\mathrm{ijk}}-\theta_{\mathrm{ik}}}{\;\sigma_{k}}\right)}^{1-I_{\mathrm{ik}}}\varphi {\left(\frac{\;Y_{\mathrm{ijk}}-\theta_{\mathrm{ik}}-\gamma_{\mathrm{ik}}{\left(t_{\mathrm{ij}}-\tau_{\mathrm{ik}}\right)}^{+}}{\;\sigma_{k}}\right)}^{I_{\mathrm{ik}}}$$

where Y denotes the set of values $Y_{\mathrm{ijk}}$, t the set of screening times $t_{\mathrm{ij}}$ for each patient, and φ is the standard normal probability density function.

A key feature of the Bayesian hierarchical model that we adopt in this paper is the statistical dependence among the levels of different biomarkers, following the methodology proposed by.^17^ This element is now compared to earlier work in.^7^, ^15^, ^16^ Dependence is explicitly introduced for the binary indicators $I_{i}={\left\{I_{\mathrm{ik}}\right\}}_{k=1,\ldots ,K}$, which are assumed to form a Markov random field (MRF). Their joint probability mass function (pmf) has the form

(2) $$\;P\left(I_{i}\right)\propto \exp\left\{\mu_{I}\sum_{k=1}^{K}I_{\mathrm{ik}}+\eta_{I}\left(I_{i}^{T}RI_{i}\right)\right\}$$

where **R** is an upper triangular matrix weighted by a coupling coefficient $\eta_{I}$. The parameter $\mu_{I}$ controls the sparsity of the model, given that not all biomarkers may increase during the onset of disease. From Expression (2), it follows that the probability of a change point in the level of the biomarker k for patient i given all the other markers is

(3) $$\;P\left\{I_{\mathrm{ik}}\;|\;I_{\mathrm{ik}\prime }:k\neq k^{\prime }\;\right\}=\frac{\exp\;\left\{I_{\mathrm{ik}}F\left(I_{\mathrm{ik}}\right)\right\}}{1+\exp\;\left\{F\left(I_{\mathrm{ik}}\right)\right\}}$$

where $F\;\left(I_{\mathrm{ik}}\right)=\mu_{I}+\eta_{I}\sum_{k\neq k\prime }I_{\mathrm{ik}\prime }$. According to Equation (3), a change point in one biomarker, for example $I_{\mathrm{ik}}=1$, implies an increase of the probability of having a change point in the remaining biomarkers whenever $\eta_{I}>0$. If we set $\eta_{I}=0$, then the biomarkers are independent (decoupled), and the probability of a change point for each single biomarker is reduced to a Bernoulli distribution with a mean parameter of $1/\left(1+\exp\left(-\mu_{I}\right)\right)$.

The mean level for the *i*‐th patient and *k*‐th biomarker is assumed Gaussian, $\theta_{\mathrm{ik}}∼N\left(\mu_{\mathrm{\theta k}},\sigma_{\mathrm{\theta k}}^{2}\right)$, where $\mu_{\mathrm{\theta k}}$ is itself Gaussian,

(4) $$\;\mu_{\mathrm{\theta k}}∼N\left(\mu_{0k},\sigma_{0k}^{2}\right)$$

The variance $\sigma_{\mathrm{\theta k}}^{2}$ an inverse gamma distribution,

(5) $$\;{\;\sigma }_{\mathrm{\theta k}}^{2}∼\mathrm{IG}\left(a_{\mathrm{\theta k}},b_{\theta_{k}}\right)$$

The hyperparameters $\left(\mu_{0k},\sigma_{0k}^{2},a_{\mathrm{\theta k}},b_{\mathrm{\theta k}}\right)$ in (4) and (5) are assumed deterministic. The binary indicators $I_{i}$ follow a Markov random field distribution, Equation (2), which we denote as $\;I_{i}∼\mathrm{MRF}\left(\pi \right),$ where $\pi =\left(\mu_{I},\eta_{I}\right)$. Following,^4^, ^7^, ^16^ approximately 15% of the patients with ovarian cancer do not show an increment in CA125 levels. In the absence of coupling, we assumed that the logistic transformation of $\mu_{I}$ follows a Beta prior distribution with a mean of 0.85 and a standard deviation of 0.05. This accounts for the proportion of cases we expect to exhibit a change point. In this work, we assume that all the biomarkers under consideration follow the same rate. Similarly, we assumed a Beta prior distribution for the parameter $\eta_{I}$, as in.^17^

(6) $$\exp\left(\mu_{I}\;\right)/\left(1+\exp\left(\mu_{I}\;\right)\right)∼\text{Beta}\left(p_{1},p_{2}\right)$$

(7) $$\eta_{I}∼\text{Beta}\left(p_{3},p_{4}\right)$$

Individual random effects for the rate log($\gamma_{ik}$) are assumed to follow an independent normal distribution log($\gamma_{ik}$) ∼ $N\left(\mu_{\mathrm{\gamma k}},\sigma_{\mathrm{\gamma k}}^{2}\right)$ where

(8) $$\;\mu_{\mathrm{\gamma k}}∼N\left(\mu_{1k},\sigma_{1k}^{2}\right)$$

(9) $$\;\sigma_{\mathrm{\gamma k}}^{2}∼\mathrm{IG}\left(a_{\mathrm{\gamma k}},b_{\mathrm{\gamma k}}\right)$$

The individual change point $\tau_{\mathrm{ik}}$ is modeled as a truncated normal distribution as in,^7^

(10) $$\;\tau_{\mathrm{ik}}∼{\mathrm{TN}}_{\left[d_{i}-\tau^{*},d_{i}\right]}\left(d_{i}-\mu_{\mathrm{\tau k}},\sigma_{\mathrm{\tau k}}^{2}\right)$$

where the mean is centered at $\mu_{\mathrm{\tau k}}=2$ years and the variance is $\sigma_{\mathrm{\tau k}}^{2}=0.75$. The distribution is truncated at $\left[d_{i}-\tau^{*},d_{i}\right]$, reflecting the preclinical duration, which is assumed to be of $\tau^{*}=5$ years.

Finally, the conditional variance of the *k*‐th biomarker level, $\sigma_{k}^{2}$ is assumed to follow an inverse gamma prior distribution $\mathrm{IG}\;\left(a,b\right)$ as in.^15^, ^16^

From the description above, we define by $K=\left\{\mu_{\mathrm{\theta k}},\mu_{\mathrm{\gamma k}},\mu_{I},\eta_{I},\sigma_{\mathrm{\theta k}}^{2},\sigma_{\mathrm{\gamma k}}^{2},\sigma_{k}^{2}\right\}$ as the set of parameters specific to each biomarker k. For a given set of observations **Y =** $\left\{Y_{\mathrm{ijk}}\right\}$, the likelihood of the parameters K and S can be readily obtained from Equation (1). Thus, the likelihood function $L\left(K,S\right)$ is Equation (1) for a given set of observations **Y**, that is

(11) $$\;L\left(K,S\right)=\prod_{i=1}^{n_{0}}\prod_{k=1}^{K}\prod_{j=1}^{T_{i}}\varphi \left(\frac{\;Y_{\mathrm{ijk}}-\theta_{\mathrm{ik}}}{\;\sigma_{k}}\right)\;\times \prod_{i=n_{0}+1}^{N}\prod_{k=1}^{K}\prod_{j=1}^{T_{i}}\varphi {\left(\frac{\;Y_{\mathrm{ijk}}-\theta_{\mathrm{ik}}}{\;\sigma_{k}}\right)}^{1-I_{\mathrm{ik}}}\varphi {\left(\frac{\;Y_{\mathrm{ijk}}-\theta_{\mathrm{ik}}-\gamma_{\mathrm{ik}}{\left(t_{\mathrm{ij}}-\tau_{\mathrm{ik}}\right)}^{+}}{\;\sigma_{k}}\right)}^{I_{\mathrm{ik}}}$$

If we let $P_{0}\left(K,S\right)$ denote the a priori probability of the model parameters, as described through Equations ((2), (3), (4), (5), (6), (7), (8), (9)), the a posteriori probability distribution of K and S given the data **Y** has the form

(12) $$\;P_{Y}\left(K,S\right)\propto L\left(K,S\right)P_{0}\left(K,S\right)$$

This posterior distribution contains all the statistical information relevant for the model. Below, we discuss methods for the numerical approximation of $P_{Y}\left(K,S\right)$. The hyperparameter values are chosen following clinical considerations discussed in^7^, ^15^, ^16^ (Table S1).

#### 2.1.2 Procedure

The posterior distribution of all the unknown parameters can be approximated using a Markov chain Monte Carlo (MCMC) algorithm, following the procedure proposed and described in full detail in.^17^ Here, we highlight the main considerations for the iteration process. First, the choice between the types of sampling is based on whether the full conditional distributions can be easily calculated or not. For the biomarker‐specific parameters, the posterior distribution for the subset of parameters $\left\{\sigma_{k}^{2},\mu_{\mathrm{\theta k}},\sigma_{\mathrm{\theta k}}^{2},\mu_{\mathrm{\gamma k}},\sigma_{\mathrm{\gamma k}}^{2}\right\}$ can be obtained using Gibbs sampling at each step of the iteration. In addition, {$\mu_{I},\eta_{I}$} are determined from Metropolis–Hasting sampling.

For the subject‐specific parameters, $\theta_{\mathrm{ik}}$, we use a Gibbs sampler. To get draws from the full conditionals of $I_{\mathrm{ik}}$, $\gamma_{\mathrm{ik}}$, and $\tau_{\mathrm{ik}}$, we use a reversible‐jump step.^18^ This comes from the construction of the change‐point parameter: $I_{\mathrm{ik}}$ provides either $\theta_{\mathrm{ik}}$ for $I_{\mathrm{ik}}=0$ (for i=1,2,…,N) or {$\theta_{\mathrm{ik}}$, $\gamma_{\mathrm{ik}}$, $\tau_{\mathrm{ik}}$} for $I_{\mathrm{ik}}=1$ (for i = $n_{0}+1$, …, N).

##### Initialization

For each parameter in K, we draw an initial sample from its a priori distribution (similarly, for each patient and each parameter in S). We initialize the MCMC iteration by sampling from the priors of the model parameters (Table S1). Then, we estimate the posterior accordingly using MCMC as described above in this subsection.

##### Iteration

In this paper, we generate two independent chains, each with different initial values, to assess the convergence to the same stationary distribution for each unknown parameter using trace plots. We also ensured convergence using the Gelman–Rubin statistic. For each chain and unknown parameter, we simulate 40,000 samples with a burn‐in period fixed at 5000 samples. The remaining samples from the two chains are combined to generate 70,000 samples in total. These were used for the calculation of the average of the joint probabilities to get an estimate of $P\left(\left(Y_{i1k},\ldots ,Y_{\mathrm{ijk}}\right)\;|\;o_{i}\right)$ for each patient at each screening time and biomarker *k*. Here, $o_{i}=\left\{0,1\right\}$ indicates whether the patient i is a control or has ovarian cancer.

##### Screening

The screening methodology in a $N^{\prime }$ patient from a testing cohort is based on the computation of the posterior probability of ovarian cancer $o_{N^{\prime }}$, $P\left(o_{N^{\prime }}=1|Y_{N^{\prime }}\right)$. The variable $Y_{N^{\prime }}$ denotes the longitudinal time series of different biomarkers for the patient up to time $t_{\mathrm{ij}}$, that is, the sequence $Y_{N\prime }=\left\{Y_{N\prime \left(j\prime ,k\right)}:j^{\prime }=1,2,\ldots ,j;k=1,2,\ldots ,K\right\}.$

(13) $$\;P\left(o_{N^{\prime }}=1|Y_{N^{\prime }}\right)=\frac{P\left(Y_{N^{\prime }}|o_{N^{\prime }}=1\right)P\left(o_{N^{\prime }}=1\right)}{\sum_{i\in \left\{0,1\right\}}P\left(Y_{N^{\prime }}|o_{N^{\prime }}=i\right)P\left(o_{N^{\prime }}=i\right)}$$

where $P\left(o_{N^{\prime }}\right)$ is the prior prevalence estimated from population data (Annual Incidence of Ovarian Cancer in the United Kingdom by 5‐year age group, 2016–2018^19^), and $\;P\left(Y_{N^{\prime }}|o_{N^{\prime }}\right)$ is estimated from the posterior predictive distribution for the $N^{\prime }$ patient at each screening time using the training data of N patients. Algorithm implementation was developed in RStudio, using the supporting codes provided by.^17^

### 2.2 Recurrent neural networks

Each subject, either a patient with ovarian cancer or a control, has a sequence of different measurements of biomarkers (CA125, HE4, and glycodelin) taken at different ages. For the *i*‐th patient, the biomarker level k=1,2,…,K is expressed by the sequence $Y_{i1k},Y_{i2k},\ldots ,Y_{iT_{i}k}$. These measurements are collected at ages $t_{i1},t_{i2},\ldots ,t_{iT_{i}}$, for all k.

We propose to use recurrent neural networks (RNNs) for the prediction of ovarian cancer using longitudinal observations (Figure S2), in line with our previous study.^15^ We used the long short‐term memory (LSTM) architecture,^20^, ^21^, ^22^, ^23^ which is a special form of RNNs that can both learn long‐term dependencies and control the flow of information to be passed from one time step to the next by means of gate units.^24^ The LSTM approach offers a significant advantage in addressing the vanishing gradient problem, which is common in traditional RNNs, making it generally more stable during training. It may suffer from computational complexity and potential overfitting, especially when dealing with small datasets. The latter, however, was mitigated by employing cross‐validation and dropout, a regularization technique.

More precisely, the LSTM equations can be written as follows,^20^, ^24^

(14) $$c_{\mathrm{ijk}}=f_{\mathrm{ijk}}⨀c_{\mathrm{ij}-1k}+i_{\mathrm{ijk}}⨀\tilde{\;c_{\mathrm{ijk}}}$$

(15) $$\;h_{\mathrm{ijk}}=o_{\mathrm{ijk}}⨀\tanh\left(c_{\mathrm{ijk}}\right)$$

where $c_{\mathrm{ijk}}$ and $h_{\mathrm{ijk}}$ denote the cell state and hidden state for patient i, time step j, and biomarker k, respectively; ⨀ denotes point‐wise multiplication. The cell state and hidden state at a fixed time step are given by vectors of size 1×H, where H is the number of hidden neurons.

The cell state of the LSTM at each time step is controlled by input and forgetting mechanisms using the equations shown below. That is, the forget gate unit $f_{\mathrm{ijk}}$ modulates the effect of the cell state of the previous step $\;c_{\mathrm{ij}-1k}$. Similarly, the external input gate $i_{\mathrm{ijk}}$ weights the contribution of the candidate cell via $\tilde{\;c_{\mathrm{ijk}}}$. Finally, the memory information in the hidden state is controlled by the output gate $o_{\mathrm{ijk}}$.

The so‐added gates $\tilde{\;c_{\mathrm{ijk}}}$, $i_{\mathrm{ijk}}$, $f_{\mathrm{ijk}}$, and $o_{\mathrm{ijk}}$ are defined by the following equations.

(16) $$\;\tilde{c_{\mathrm{ij}k}}=\tanh\left(h_{\mathrm{ij}-1k}U_{c}+Y_{\mathrm{ijk}}W_{c}+b_{c}\right)$$

(17) $$\;i_{\mathrm{ijk}}=\sigma \left(h_{\mathrm{ij}-1k}U_{i}+Y_{\mathrm{ijk}}W_{i}+b_{i}\right)$$

(18) $$\;\;f_{\mathrm{ijk}}=\sigma \left(h_{\mathrm{ij}-1k}U_{f}+Y_{\mathrm{ijk}}W_{f}+b_{f}\right)$$

(19) $$\;o_{\mathrm{ijk}}=\sigma \left(h_{\mathrm{ij}-1k}U_{o}+Y_{\mathrm{ijk}}W_{o}+b_{o}\right)$$

where $\sigma \left(x\right)=1/\left(1+\exp\left(-x\right)\right)$ denotes the sigmoid function. The terms $W_{c},$ $W_{f}$, $W_{i}$, and $W_{o}$ denote the weight matrices, with dimension 1×H; the terms $U_{c}$, $U_{f}$, $U_{i}$, and $U_{o}$ denote the kernel matrices of size H×H; vectors $b_{c}$, $b_{f}$, $b_{i}$, and $b_{o}$ denote the bias of size 1×H. The terms W,U, and b are learned during training.

Thus, for each time step j, we obtain a temporal sequence of hidden states ($h_{i1k}$, $h_{i2k}$, …, $h_{iT_{i}k}$) corresponding to the *i*‐th subject and biomarker k. Here, the state of the network at the last step is denoted $h_{iT_{i}k}$, which corresponds to the last sample of the patient under consideration.

Next, we can concatenate the last hidden state associated to the output of each LSTM cell from the K different biomarkers. We also include the last hidden state associated to the LSTM processing the screening age $t_{\mathrm{ij}}$ as a longitudinal feature.^25^ That is,

(20) $$\;\;h_{i}=\left[h_{iT_{i}1},h_{iT_{i}2},\ldots ,h_{iT_{i}K},h_{iT_{i}0}\right]$$

where $h_{i}$ is the last hidden state for *i*‐th patient, resulting from the concatenation of the last hidden state of each one of the K biomarkers under consideration, and an additional one associated with the age of the patient $h_{iT_{i}0}$.

To define (20), we can consider multiple combinations of biomarkers to analyze how the joint interaction of them impacts the model classification performance. In this paper, whichever is the case, we always include the age of the patient in our model as a feature. The resulting vector is of size 1×($\;H_{1}+H_{2}+\cdots +H_{K}+H_{0}$), where each $H_{l}$ denotes the number of hidden neurons used for each LSTM associated with each one of the features, that is, biomarkers k=1,2,…,K and age, respectively.

The final output of the proposed model is

(21) $$\hat{o_{i}}=\sigma \left(W_{e}\tilde{h_{i}}+b_{e}\right)$$

where $\tilde{h_{i}}$ denotes the last hidden state after dropout, $W_{e}$ is a weight vector of size ($H_{1}+H_{2}+\cdots +H_{K}+H_{0}$) ×1 and $b_{e}$ is a scalar bias. We optimize the weight matrices and biases using the cross‐entropy loss.^26^

(22) $$\;L\left(o_{i},\hat{o_{i}}\right)=\frac{1}{N}\sum_{i=1}^{N}-\left(o_{i}\log\left(\hat{o_{i}}\right)+\left(1-o_{i}\right)\log\left(1-\hat{o_{i}}\right)\right)$$

where N is the number of patients, $o_{i}$ is the true label of subject i (0 for controls and 1 for cases), and $\hat{o_{i}}$ is the estimated probability of risk of ovarian cancer.

The RNNs must be adequately trained before they can be used for the classification of unknown subjects. For the training phase, we used batch gradient descent with dynamic learning rates updated through the Adam optimizer.^24^, ^27^ The hyperparameter tuning number is defined by the number of hidden neurons and dropout rate. Meanwhile, the learning rate and number of epochs are set fixed as meta‐parameters (Table S2). The weight matrix for the recurrent state is initialized by a random orthogonal matrix, while for the inputs we use a weight matrix initialized using the Glorot's scheme.^28^, ^29^ The bias of each transformation is initialized at zero.

As part of the preprocessing step, the features are standardized during training by obtaining the mean and variance for each one of them. The same scaling transformation used during training is then applied for validation. Finally, the input data for the LSTM network were masked and padded to handle variable sequence lengths.^30^, ^31^, ^32^, ^33^, ^34^, ^35^, ^36^ Deep‐learning algorithms were implemented in Python 3.8.8 using TensorFlow version 2.11.0 and Keras version 2.11.0.

### 2.3 Simulation, model selection, and evaluation

The detection scheme for each patient at each screening time is based on the soft classification of ovarian cancer using multiple correlated longitudinal biomarkers (CA125, HE4, and glycodelin). Our methodology is based on fully Bayesian screening based on change‐point models (BCP) and LSTM‐based models (RNNs).

As a preprocessing step for both methodologies, the biomarkers are transformed into the form $Y=\log\left(Z+4\right)$, where Z is a particular biomarker.^7^, ^15^, ^37^

To evaluate the screening performance in both approaches, we use stratified 5‐fold cross‐validation with two repetitions (outer loop). This approach is particularly helpful in reducing the bias in performance evaluation. In this way, each fold divides the data into one set of training data and testing data preserving the proportion between cases and controls. Our main evaluation metrics included area under the ROC curve (AUC) and sensitivity (at 90% specificity). Significance was determined using the permutation test for mean of paired differences between two models.

For the Bayesian method, we estimate the posterior distribution of the parameters at each fold using N patients in the training data. In turn, $P\left(Y_{N^{\prime }}|o_{N^{\prime }}=0\right)$ and $P\left(Y_{N^{\prime }}|o_{N^{\prime }}=1\right)$ are calculated from the posterior predictive distribution through these biomarker levels for the patient $N^{\prime }>N$ in the testing data. The probability of having ovarian cancer is then calculated using (13). As mentioned before, convergence of the MCMC chains was determined using the Gelman–Rubin statistic for each of the posterior parameters (extracted from training data).

When considering deep‐learning‐based models, for each of the 10 iterations obtained from the outer loop we perform hyperparameter tuning on hidden neurons and dropout rate for model selection. The inner loop consists of a 10‐fold cross‐validation (3 repetitions). Once the optimal set of hyperparameters is selected, we estimate the probability of having ovarian cancer on the data held‐out from training for each patient (and each longitudinal observation). We repeat this step for every outer fold. Flow chart describing the design of the study is presented in Figure S3.

### 2.4 Lead‐time analysis

Patients developing cancer show detectable preclinical elevations of biomarkers. The literature reports that patients with ovarian cancer show abnormal rise in these biomarkers approximately 3 years before diagnosis, with detectable elevations becoming apparent in the last year before diagnosis. We assess the potential value of using models based on multiple biomarkers in detecting cancer at earlier stages than it would be if diagnosed clinically.^12^, ^14^, ^38^, ^39^ In the results section, we determine the model‐based lead time of each one of the proposed screening tests. This is defined as the interval from being correctly classified as case by the diagnostic test to the actual clinical diagnosis. To discriminate patients, we detect the earliest observation per patient considered as abnormal using a threshold at 90% specificity.^40^ We then calculate the interval from this screening time point up to the time of diagnosis. This procedure is repeated over multiple outer folds in the cross‐validation procedure, from which we get different statistics for each of the models under consideration.

## 3 RESULTS

### 3.1 Data

The data consist of 224 patients with serum samples sourced from the multimodal arm of UK Collaborative Trial of Ovarian Cancer Screening (UKCTOCS, number ISRCTN22488978; NCT00058032^41^). This includes 180 controls (healthy subjects) and 44 cases (diagnosed patients). The eligible patients attended for screening and had an annual serum CA125 level measured as a baseline and transvaginal ultrasound in women as a second‐line test. Additional biomarker assays human epididymis 4 (HE4) and glycodelin (PAEP) were performed within a subset of the serial samples from the general population of the UKCTOCS trial.

The screening age range of cases is [52.0, 77.4] years with an average of 65.3 years. The dataset includes the biomarkers history per patient for up to 5 years. Out of the 44 cases, 12 cases are screened for 1 year prior to clinical diagnosis, 12 cases for 2 years, and 20 cases for up to 3–5 years. In addition, from this set of cases 10 have 2 samples, another additional 10 have 3 samples, and 24 cases have 5 samples. For controls, the screening age is within the range of [50.3, 78.8] years with an average age of 63.6 years. Each patient has four to five observations (for 2 and 178 controls, respectively).

Within the cohort of cases, 35 patients present primary invasive epithelial ovarian cancer (average age of 64.4 years within a range of [52.0, 77.4] years), 6 cases of malignant neoplasm of peritoneum (average 68.5 years within [62.5, 76.3] years), and 3 cases of primary invasive fallopian tube cancer (average 68.2 years within [64.4, 70.8] years).

The available information indicates that of the 44 cases, 16 are in the early stage (I−II) and 28 late stages (III−IV). The morphology of cancers was predominantly serous (*n* = 27 cases). Other types include papillary (*n* = 2), endometrioid (*n* = 3), clear cell (*n* = 2), carcinosarcoma (*n* = 3), carcinoma (*n* = 2), neoplasm malignant (*n* = 1), and adenocarcinoma (*n* = 4).

In addition to CA125, human epididymis 4 (HE4) and glycodelin (PAEP) were selected based on previous studies.^12^, ^14^, ^15^, ^16^ All serum samples were assayed by ELISA (enzyme‐linked immunosorbent assay). Previous reports discarded possible confounding effects of sample processing using correlation between the concentration of any of the samples and time between sample collection and spin.^14^

**FIGURE 1:**
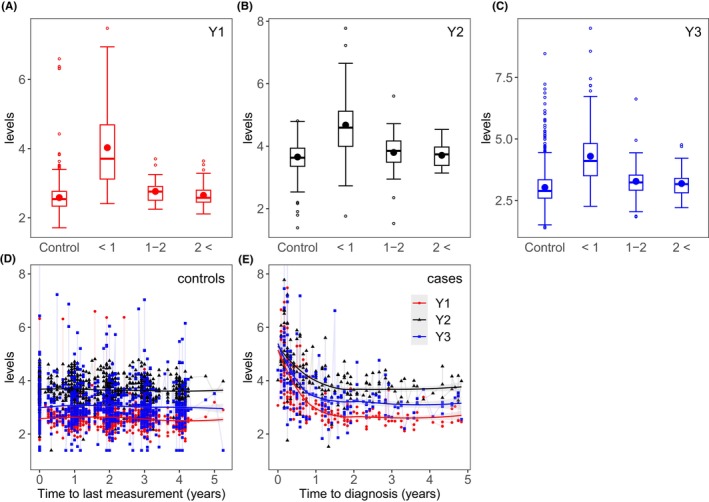
(A–C) Box plots of protein biomarker levels (Y1: CA125, Y2: HE4, and Y3: glycodelin) in controls and cases. For each panel, the cases are grouped in time ranges: year, between 1 and 2 or more than 2 years before diagnosis. (D–E) Biomarker levels in cases for CA125, HE4, and glycodelin for cases and controls, respectively. Loess curves fit has been added to depict the trend prior to clinical diagnosis. As observed, the biomarkers for case patients exhibit a significant increase within the final year. The biomarker levels have been log‐transformed in all the plots.

Longitudinal observations of biomarkers in case patients prior to clinical diagnosis are displayed in Figure 1. Loess curves were fit to reflect the mean levels over time. It is observed that for case patients the biomarker levels start to slowly rise between 1 and 2 years prior to diagnosis. This is particularly noticeable for CA125 and HE4, while for glycodelin the rise is to a limited extent. This rise becomes more pronounced within 1 year before diagnosis, in which all the biomarkers show recognizable elevations. In addition, from a qualitative perspective, the rate of increase within 1 year appears to be led by CA125 followed by glycodelin and then HE4.

### 3.2 Multivariable longitudinal models

Next, we evaluate the performance of different models in the detection of cases. The simulation studies consider multiple scenarios, including three joint multivariable screening tests that combine CA125 levels with other biomarkers (HE4 and glycodelin), as well as three single biomarker tests. More specifically, we have considered the following scenarios:

- m(1,2,3): CA125‐HE4‐glycodelin
- m(1,2): CA125‐HE4
- m(1,3): CA125‐glycodelin
- u(1): CA125
- u(2): HE4
- u(3): glycodelin

Notation m(i,j,k) (or m(i,j)) indicates a joint multivariable test that relies on the biomarkers i,j, and k; while u(i) refers to a test that uses the *i*‐th biomarker alone. The biomarkers are numbered as CA125 (1), HE4 (2), and glycodelin (3). We use two classification methodologies based on Bayesian change‐point and recurrent neural network models.

The risk of ovarian cancer is estimated for each patient's longitudinal observation starting from two visits up to all the screening period. That is, for each screening time point $t_{i}^{*}$ starting from the second visit, the model makes a prediction based on the patients' previous observations $t_{i}<t_{i}^{*}.$ Unless it is stated, we report the performance metrics using all the screening period, which implies that we estimate the risk of ovarian cancer in the last patient's screening time point.

**FIGURE 2:**
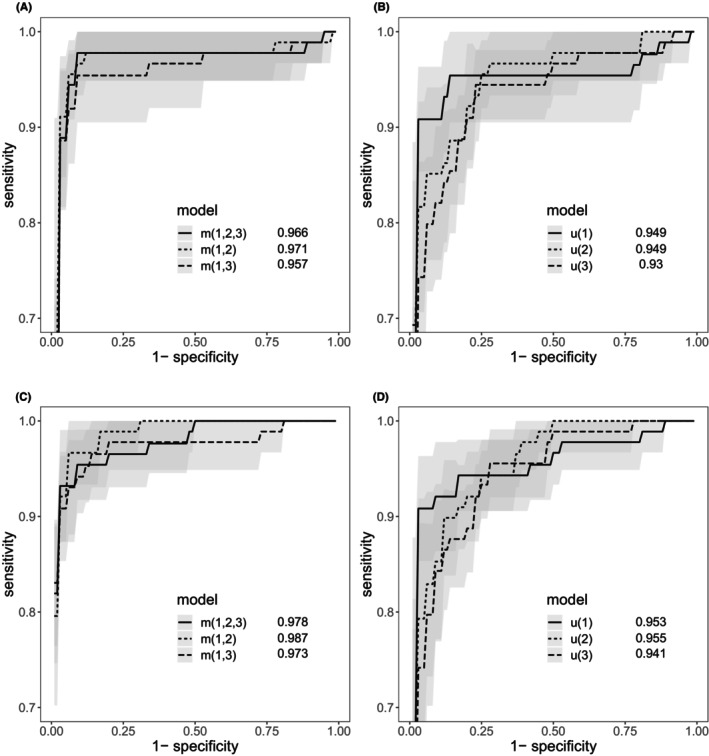
Cross‐validated ROC curves with 95% confidence intervals and AUC using models based on a (A, C) joint multivariable and (B, D) Univariate approach. Figures (A, B) correspond to Bayesian change point and (C, D) To recurrent neural networks.

Figure 2 shows the receiver operating characteristic (ROC) curve indicating the area under the curve (AUC) statistic. This provides a summary of the classification performance in each case. These results are calculated by averaging the sensitivity at a given level of specificity for each one of the outer folds obtained by cross‐validation.

The ROC curves suggest an improvement in using additional biomarkers over single biomarkers as the sensitivity of joint multivariable tests lie above the univariate ones using BCP and RNNs models. Furthermore, the sensitivity of joint multivariable models shows confidence intervals slightly reduced with respect to using single biomarkers.

From Table 1, we observe that m(1,2) (CA125 and HE4) performs substantially better among the joint multivariable models. Using BCP, AUC is of 0.971 (96.7% sensitivity). Meanwhile with RNNs, AUC equal to 0.987 (with 96.7% sensitivity). In the univariate case, u(1) (CA125) outperforms over all the other single biomarkers in terms of the sensitivity score. Using BCP, it scores 90.8% sensitivity (AUC, 0.949), while for RNNs it is 92.1% sensitivity (AUC, 0.953).

**TABLE 1:** Model performance for the detection of ovarian cancer diagnosed within 1 year: estimates of sensitivity (90% specificity) and AUC, and their 95% confidence intervals (CI) comparing different models (joint multivariable and univariate) based on cross‐validation procedure.

Model	Sensitivity	(95% CI)	*p*‐value	AUC	(95% CI)	*p*‐value
BCP						
m(1,2,3)	0.978	0.949, 1.0	**0.016**	0.966	0.941, 0.991	0.234
m(1,2)	0.967	0.934, 1.0	**0.031**	0.971	0.946, 0.996	**0.043**
m(1,3)	0.954	0.905, 1.0	0.062	0.957	0.924, 0.990	0.187
u(1)	0.908	0.853, 0.963		0.949	0.908, 0.990	
u(2)	0.851	0.761, 0.941	0.875	0.949	0.920, 0.978	0.527
u(3)	0.821	0.741, 0.901	0.934	0.930	0.905, 0.955	0.883
RNNs						
m(1,2,3)	0.954	0.917, 0.991	0.125	0.978	0.962, 0.994	**0.002**
m(1,2)	0.967	0.934, 1.0	0.062	0.987	0.979, 0.995	**0.002**
m(1,3)	0.942	0.903, 0.981	0.313	0.973	0.951, 0.995	**0.006**
u(1)	0.921	0.864, 0.978		0.953	0.928, 0.978	
u(2)	0.853	0.773, 0.933	0.938	0.955	0.935, 0.975	0.455
u(3)	0.843	0.769, 0.917	0.984	0.941	0.917, 0.965	0.872

*Note*: One‐sided *p*‐values are determined from permutation test on the difference of mean AUC (or sensitivity) between diagnostic tests with respect to our baseline (CA125). Bold values indicate p < 0.05 considered as statistically significant.

The combination of CA125 with HE4 provides improvement of the AUC score and sensitivity over the univariate tests using the proposed methodologies. To test the significance of such improvement, we used the permutation test for mean of paired differences with respect to CA125 for these two metrics using BCP and RNNs. For the AUC, we get the one‐sided *p* = 0.043 and p=0.002, respectively. Meanwhile, for the sensitivity, we obtain 0.031 and 0.062.

The second best‐performing scheme is m(1,2,3) (CA125, HE4, glycodelin). In a similar way, the permutation test for the AUC provides *p* = 0.234 and 0.002, while for the sensitivity *p* = 0.016 and 0.125, using BCP and RNNs, respectively.

In Table 2a, using case patients only, we build a contingency table, to compare the best two diagnostic tests (joint multivariable and univariate, respectively) based on data from all the outer folds. The tests used a threshold set at 90% specificity. As observed, the joint multivariable model m(1,2) (CA125 and HE4) attains higher sensitivity than the reference standard, CA125. This holds for both RNNs and BCP methodologies. Applying a McNemar test is unfeasible in this case as the power is dependent on discordant pair sample size, which in our case is rather small. However, the detection rate in addition to the results shown above suggests that the combination of CA125 and HE4 has potential to improve current tests based on CA125 alone.

**TABLE 2:** (A) Results obtained from contingency tables at 90% specificity level. The tests are either based on recurrent neural networks (RNNs) or Bayesian change‐point models (BCP). Estimations are based on the cross‐validation procedure. (B) Number of correctly diagnosed and missed cases.

		BCP m(1,2)		RNNs m(1,2)	
		Diagnosed	Missed	Diagnosed	Missed
**(A)**					
u(1)	Diagnosed	80	0	81	0
u(1)	Missed	5	3	4	3

	BCP		RNNs	
	Diagnosed	Missed	Diagnosed	Missed
**(B)**				
m(1,2,3)	86	2	84	4
m(1,2)	85	3	85	3
m(1,3)	84	4	83	5
u(1)	80	8	81	7
u(2)	75	13	75	13
u(3)	72	16	74	14

*Note*: For the calculation, we detect the earliest observation per patient considered as abnormal using a threshold at 90% specificity. Values correspond to the total number over all outer folds.

**FIGURE 3:**
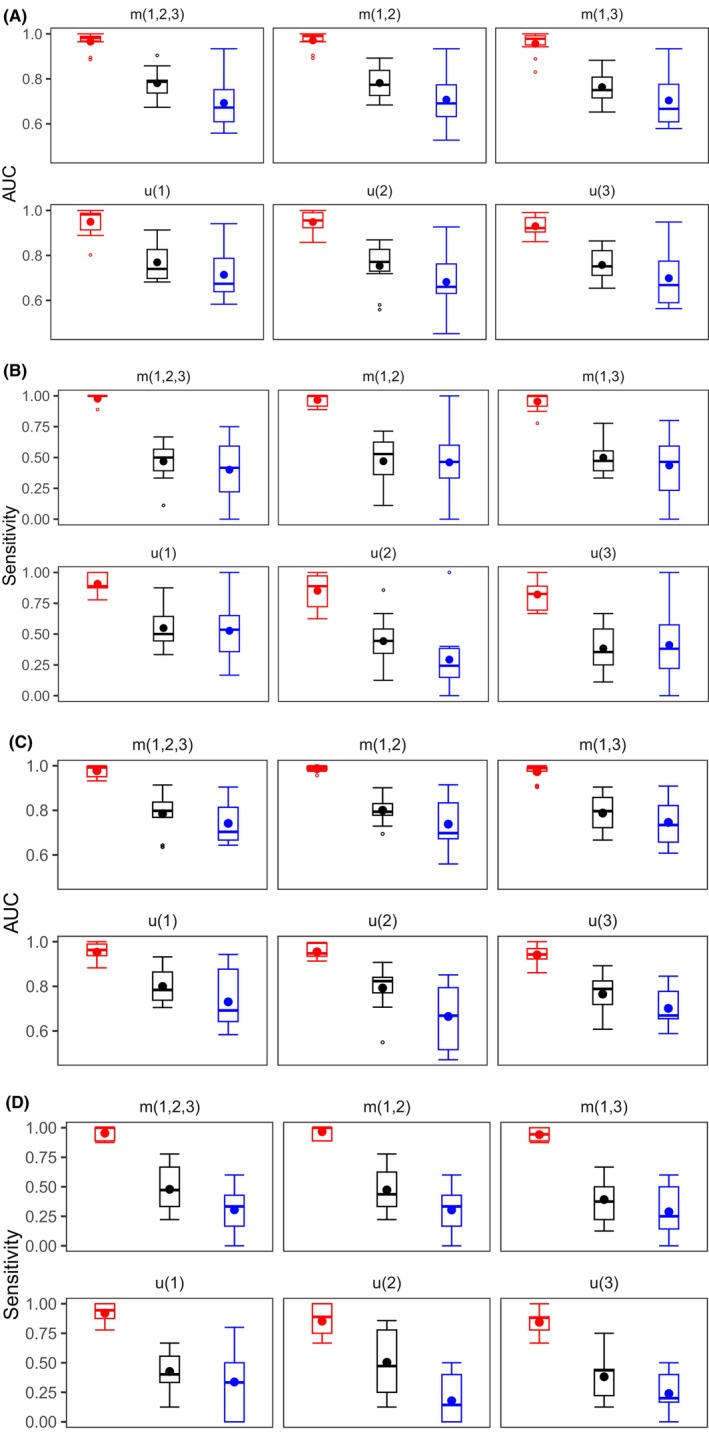
Each box plot displays estimates of (A, C) AUC and (B, D) sensitivity (90% specificity) comparing changes at different models (joint multivariable m(1,2,3), m(1,2), and m(1,3), and univariate u(1), u(2), and u(3)) and time periods (left to right: all screening period, 1 and 2 years before diagnosis). Each plot results from the cross‐validation procedure. Figures (A, B) correspond to Bayesian change point and (C, D) to recurrent neural networks.

Next, we study the ability of our algorithms to detect the ovarian cancer at earlier stages.^12^, ^14^ In Figure 3, we show the summary statistics of the AUC and sensitivity (90% specificity) estimated by screening the last time point available per patient using the complete longitudinal history, 1 and 2 years prior to clinical diagnosis. We observe that both metrics decrease as we screen patients with biomarker levels taken at earlier times.

Before 1 year of diagnosis, CA125–HE4 ranks as the best joint multivariable model. Using BCP, AUC scores 0.782 (with 47.0% sensitivity), whereas for RNNs, AUC scores 0.8 (47.3% sensitivity). In the univariate case, CA125 has AUC equal to 0.769 (54.9% sensitivity) using BCP, and AUC of 0.799 (42.6% sensitivity) for RNNs. Two years before diagnosis, CA125 alone provided higher sensitivity than any other model. Using BCP, we get 52.7% sensitivity and AUC equal to 0.714.

The estimated mean lead time based on joint multivariable tests spanned from 1.6 to 1.9 years, while median lead time from 1.4 to 1.8 years prior to diagnosis in comparison with the estimated range based on CA125 alone, Table 3. No multivariable algorithm significantly outperformed CA125 using both BCP and RNNs.

**TABLE 3:** Summary statistics of model‐based (joint multivariable and univariate) lead time for the detection of ovarian cancer at 90% specificity. Estimations are based on cross‐validation procedure.

Model	Mean lead time (95% CI)		Median lead time (95% CI)		Min lead time (95% CI)		Max lead time (95% CI)	
BCP								
m(1,2,3)	1.939	1.723,2.155	1.783	1.489, 2.077	0.508	0.367, 0.649	3.967	3.538, 4.396
m(1,2)	1.912	1.687,2.137	1.629	1.386, 1.872	0.567	0.402, 0.732	3.883	3.379, 4.387
m(1,3)	1.882	1.649,2.115	1.608	1.390, 1.826	0.542	0.313, 0.771	3.892	3.429, 4.355
u(1)	1.917	1.633, 2.201	1.704	1.426, 1.982	0.625	0.392, 0.858	3.467	2.942, 3.992
u(2)	1.575	1.410, 1.740	1.387	1.269, 1.505	0.350	0.203, 0.497	3.158	2.623, 3.693
u(3)	1.685	1.524, 1.846	1.512	1.351,1.673	0.458	0.270, 0.646	3.533	2.886, 4.180
RNNs								
m(1,2,3)	1.597	1.283, 1.911	1.392	1.045, 1.739	0.433	0.286, 0.580	3.617	2.882, 4.352
m(1,2)	1.768	1.558, 1.978	1.504	1.290, 1.718	0.517	0.345, 0.689	4.050	3.529, 4.571
m(1,3)	1.604	1.288, 1.920	1.375	1.054, 1.696	0.483	0.281, 0.685	3.492	2.835, 4.149
u(1)	1.877	1.440, 2.314	1.579	1.197, 1.961	0.758	0.446, 1.070	3.700	2.983, 4.417
u(2)	1.672	1.315, 2.029	1.517	1.207, 1.827	0.367	0.202, 0.532	3.525	2.714, 4.336
u(3)	2.016	1.673, 2.359	1.687	1.305, 2.069	0.725	0.425, 1.025	4.017	3.323, 4.711

In line with the results above, there is a strong indication that the combination of CA125 and HE4 increases the classification performance over CA125 alone. This is based on AUC and sensitivity at 90% specificity. Furthermore, we also find that this combination outperforms over CA125 and all other alternatives based on the ratio between correctly diagnosed cases and missed ones by the algorithm, Table 2b. Finally, its mean lead time is close to 2 years.

Overall, our results emphasize once again the benefit of using HE4 as a complementary biomarker that deserves further evaluation for the improvement of early detection of ovarian cancer compared with CA125 alone.

## 4 DISCUSSION

The present study addresses two distinct issues—methodological, related to the integration of multiple longitudinal biomarkers into a single model, and practical, concerning the enhancement of performance rates in ovarian cancer detection through longitudinal data analysis.

We compared two longitudinal algorithms allowing the integration of more than one biomarker—a Bayesian change‐point model and a LSTM architecture of the recurrent neural networks approach. Our findings show that the combination of longitudinal CA125 and HE4 levels outperforms the CA125‐only model with both the change‐point model and the LSTM method, highlighting the complementary nature of HE4. The multimarker model did not improve the lead time but provided higher area under the ROC curve (AUC) and sensitivity at a fixed specificity, potentially improving early cancer detection. Importantly, this work illustrates the advantage of the methodology for the simultaneous analysis of multiple longitudinal biomarkers that may prove useful in any scenario where such biomarkers emerge, particularly in early cancer detection.

Previous research in ovarian cancer has mainly concentrated on multiple biomarkers at a single time point or on longitudinal CA125, the best‐performing individual biomarker.^8^, ^37^, ^40^ However, the UKCTOCS trial demonstrated that monitoring CA125 alone does not provide significant mortality benefits.^5^ Consequently, there is an urgent need to explore additional potential biomarkers that could improve detection rates. Our previous work focused on the analysis of longitudinal HE4, CA72‐4, and anti‐TP53.^12^ The findings suggested that these biomarkers offer limited additional value to longitudinal CA125. However, the study was limited to using only the MMT approach thus emphasizing the significance of the present work.

The primary limitation of our study is the small sample size, which we attempted to address through the nested cross‐validation approach. Both cancer cases and controls were randomly selected from the complete dataset. Since the UKCTOCS was a randomized trial, this selection process should mitigate potential confounding factors and biases. Another limitation is that only two approaches have been tested in this study, and the biomarker panel comprised only three proteins. We focused on currently available approaches for analyzing time‐series biomarker data in the cancer setting, utilizing the available dataset. While we incorporated some of the most prominent biomarkers, it is important to note that future studies may achieve improved performance with a larger panel of biomarkers and potentially new statistical and AI methodologies. The primary strength of our work is the use of the unique dataset obtained from the UKCTOCS trial.

In conclusion, our investigation provides evidence of enhanced performance using a combination of longitudinal biomarkers compared with the best individual longitudinal CA125. To the best of our knowledge, this is the first investigation in which statistical and artificial intelligence (AI) approaches have been employed and compared for the analysis of multiple longitudinal biomarkers, not only in the context of ovarian cancer but also in other healthcare settings. The findings from the current work could be applied to facilitate early detection, risk stratification, and the prevention and treatment of various diseases. The enhanced early detection capabilities of the CA125–HE4 multimarker model hold the potential to significantly improve patient outcomes by enabling timely diagnosis, assisting healthcare providers in more accurate clinical decision‐making, and informing policymakers in shaping effective strategies for ovarian cancer screening and management.

These findings now warrant blinded validation in a larger longitudinal sample set to assess the potential for early detection in ovarian cancer screening.

## AUTHOR CONTRIBUTIONS

**Luis Abrego:** Formal analysis (equal); visualization (equal); writing – original draft (equal); writing – review and editing (equal). **Alexey Zaikin:** Formal analysis (equal); funding acquisition (supporting); writing – original draft (equal); writing – review and editing (equal). **Ines P. Marino:** Formal analysis (equal); visualization (equal); writing – original draft (equal); writing – review and editing (equal). **Ian Jacobs:** Conceptualization (equal); methodology (equal); writing – review and editing (equal). **Usha Menon:** Conceptualization (equal); data curation (equal); supervision (equal); writing – review and editing (equal). **Aleksandra Gentry‐Maharaj:** Conceptualization (equal); data curation (equal); project administration (equal); writing – original draft (equal); writing – review and editing (equal). **Oleg Blyuss:** Conceptualization (equal); formal analysis (equal); funding acquisition (lead); methodology (equal); supervision (equal); writing – original draft (equal); writing – review and editing (equal). **Mikhail I. Krivonosov:** Writing – original draft (equal); writing – review and editing (equal).

## CONFLICT OF INTEREST STATEMENT

U.M. and I.J. declare financial interest through UCL Business and Abcodia Ltd in the third‐party exploitation of clinical trial biobanks, which have been developed through research at UCL. The remaining authors declare no conflict of interest.

## ETHICS APPROVAL AND CONSENT TO PARTICIPATE

This nested case–control study within UKCTOCS was approved by the Joint UCL/UCLH Research Ethics Committee A (Ref. 05/Q0505/57). Written informed consent was obtained from donors, and no data allowing identification of patients were provided. The study was performed in accordance with the Declaration of Helsinki.

## Supporting information


Data S1.

## Data Availability

Raw assay data, excepting CA125, are available upon request.
