# Supplementary material for: Bayesian and deep‐learning models applied to the early detection of ovarian cancer using multiple longitudinal biomarkers
Source: Cancer Med. 2024 Apr 10;13(7):e7163. doi: 10.1002/cam4.7163 (PMC11004913; doi:10.1002/cam4.7163)
Supplement: Supplementary file 1 — Data S1. [file CAM4-13-e7163-s001.docx]

Bayesian and deep-learning models applied on the early detection of ovarian cancer using multiple longitudinal biomarkers

**Supplementary Information
Supplementary Methods**

We show the model diagrams related to our methods for classification. The model parameters for the Bayesian-change point detection algorithm and deep learning model are also included. In agreement with the notation of the manuscript, each patient is labelled by $i = 1, 2, . . . , n_{0}, n_{0} + 1, . . . , N$, where$n_{0}$ is the number of controls and the remaining subjects account as cases.

Each biomarker is indexed by $k =1, 2, . . . , K.$ Each patient $i$ has a set of screening visits$t_{ij}$ from zero up to time of last measurement, where $j = 1, 2, . . . , T_{i}$.

## Joint multivariable fully Bayesian model


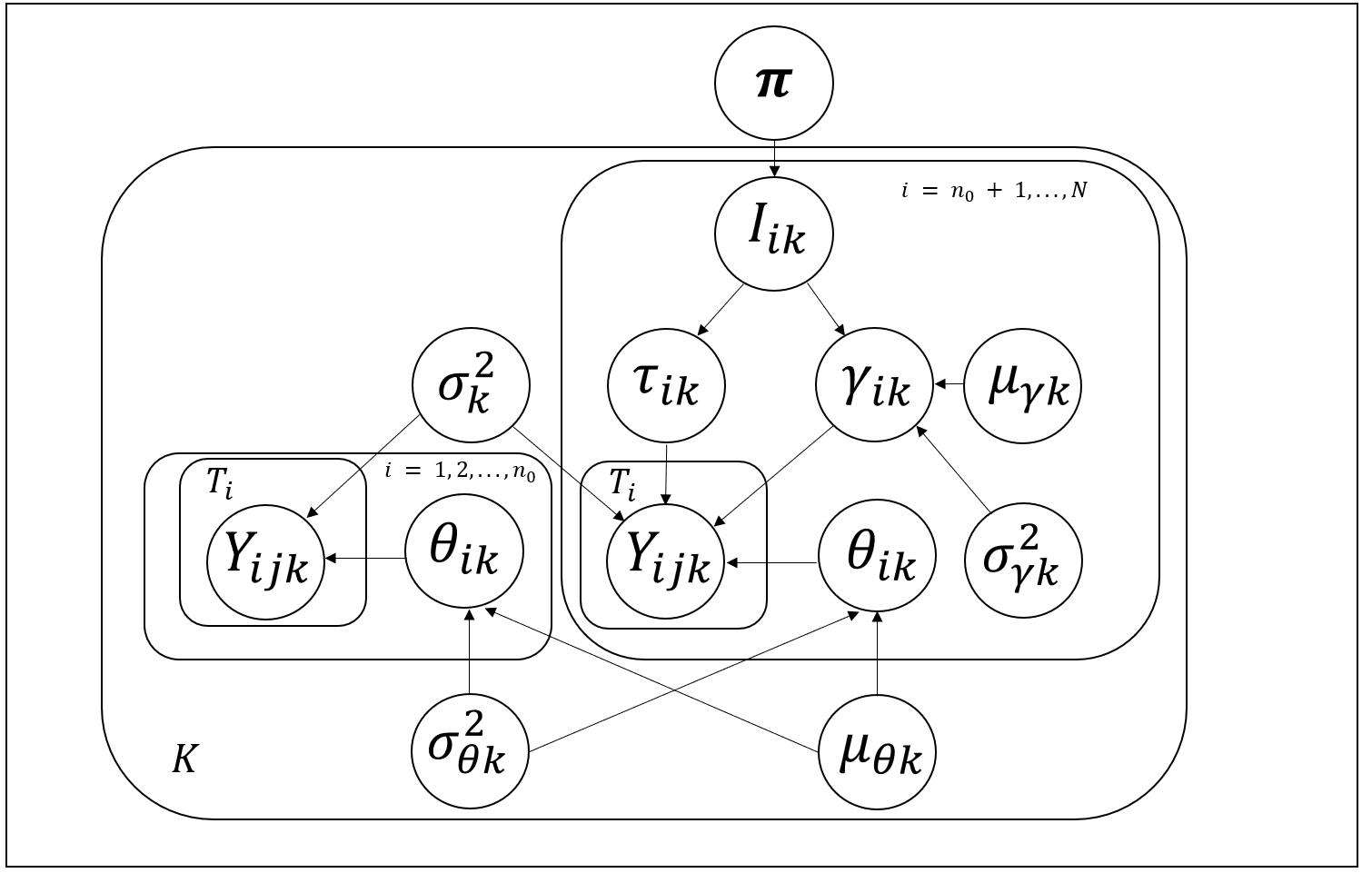


**Supplementary figure 1**: Hierarchical Bayesian model, section 3.1.1, Eqs. (1) – (10).

| **Priors** | |
| --- | --- |
| **Subject specific** | **Biomarker specific** |
| ${\theta_{ik}}^{(0)}\sim N\left( {\mu_{\theta k}}^{(0)},{\sigma_{\theta k}^{2}}^{(0)} \right)$ | ${\mu_{\theta k}}^{(0)}\sim N\left( 2. 75,1.0 \right)$ |
| ${\mathbf{I}_{i}}^{(0)} \sim MRF({\mu_{I}}^{(0)}, {\eta_{I}}^{(0)})$ | ${\sigma_{\theta k}^{2}}^{(0)}\sim IG\left( 2.04, 0.065 \right)$ |
| log(${\gamma_{ik}}^{(0)}$) ∼ $N\left( {\mu_{\gamma k}}^{(0)},{\sigma_{\gamma k}^{2}}^{(0)} \right)$ | ${\mu_{\gamma k}}^{(0)}\sim N\left( 1.1,0.1 \right)$ |
| ${\tau_{ik}}^{(0)}\sim\text{TN}_{\left[ d_{i}-5,d_{i} \right]}\left( d_{i}-2.0,{0. 75}^{2} \right)$ | ${\sigma_{\gamma k}^{2}}^{(0)}\sim IG\left( 2.2, 0.12 \right)$ |
| **MRF parameters** | ${\sigma_{k}^{2}}^{(0)}\sim IG\left( 2.05,0.1 \right)$ |
| $exp ({\mu_{I}}^{(0)})/ (1 + exp({\mu_{I}}^{(0)} )) \sim Beta(42.5, 7.5)$ |  |
| ${\eta_{I}}^{(0)}\sim Beta(5.0, 45.0)$ |  |

**Supplementary table 1.** Initialization of MCMC chain is set by sampling from the prior of model parameters.

**Recurrent Neural Networks**


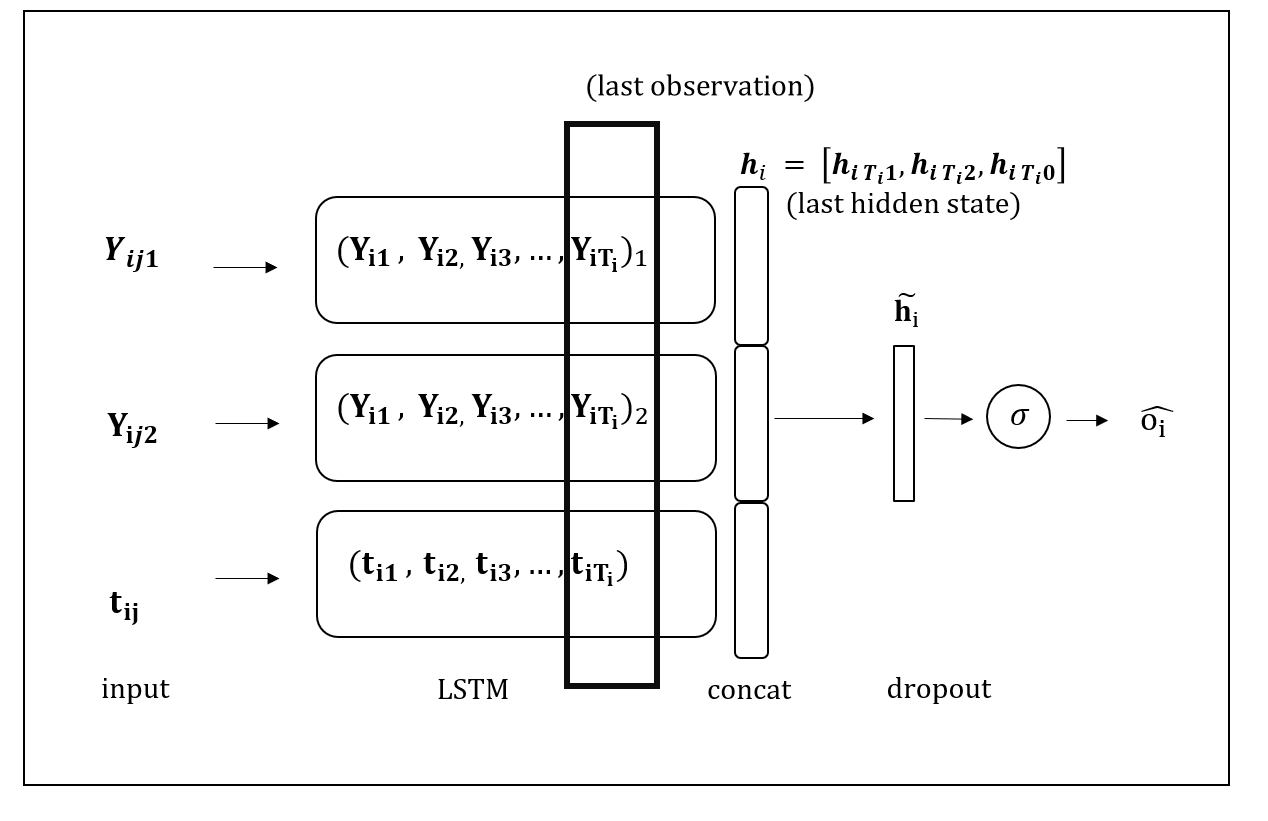


**Supplementary figure 2**: Multi-stream classification using LSTM cells. Eqs. (14) – (21). The number of biomarkers has been set to two for simplicity.

| **Model** | **Hyperparameter search range** |
| --- | --- |
| m(1,2,3) | hidden units: (8,8,2,2), (8,8,4,2), (8,8,6,2)  learning rate: 0.005  epochs: 260  dropout: 0.1, 0.2, 0.3 |
| m(1,2) | hidden units: (8,8,2), (8,10,2), (8,12,2)  learning rate: 0.005  epochs: 260  dropout: 0.1, 0.3, 0.5 |
| m(1,3) | hidden units: (8,10,2), (8,12,2), (8,14,2)  learning rate 0.005  epochs: 260  dropout: 0.1, 0.3, 0.5 |
| u(1) | hidden units: (2,2), (4,2), (6,2)  learning rate: 0.004  epochs: 260  dropout: 0.1, 0.2, 0.3 |
| u(2) | hidden units: (4,2), (6,2), (8,2)  learning rate: 0.004  epochs: 260  dropout: 0.1, 0.2, 0.3 |
| u(3) | hidden units: (8,2), (10,2), (14,2)  learning rate: 0.004  epochs: 260  dropout: 0.1, 0.2, 0.3 |

**Supplementary table 2.** Hyperparameters used for LSTM-based model. Model selection based on 10-fold cross-validation (3 repetitions). Each optimal combination is tested on the held-out data set from set by the outer loop (10 iterations using 5-fold cross-validation with two repetitions). Note that the hidden units are represented here by tuples. Each dimension corresponds to the number of hidden units associated to each biomarker’s LSTM cell. The last dimension of the tuple is associated with the sequence of ages at which the sample was taken, as described in methods, Section 3.2.


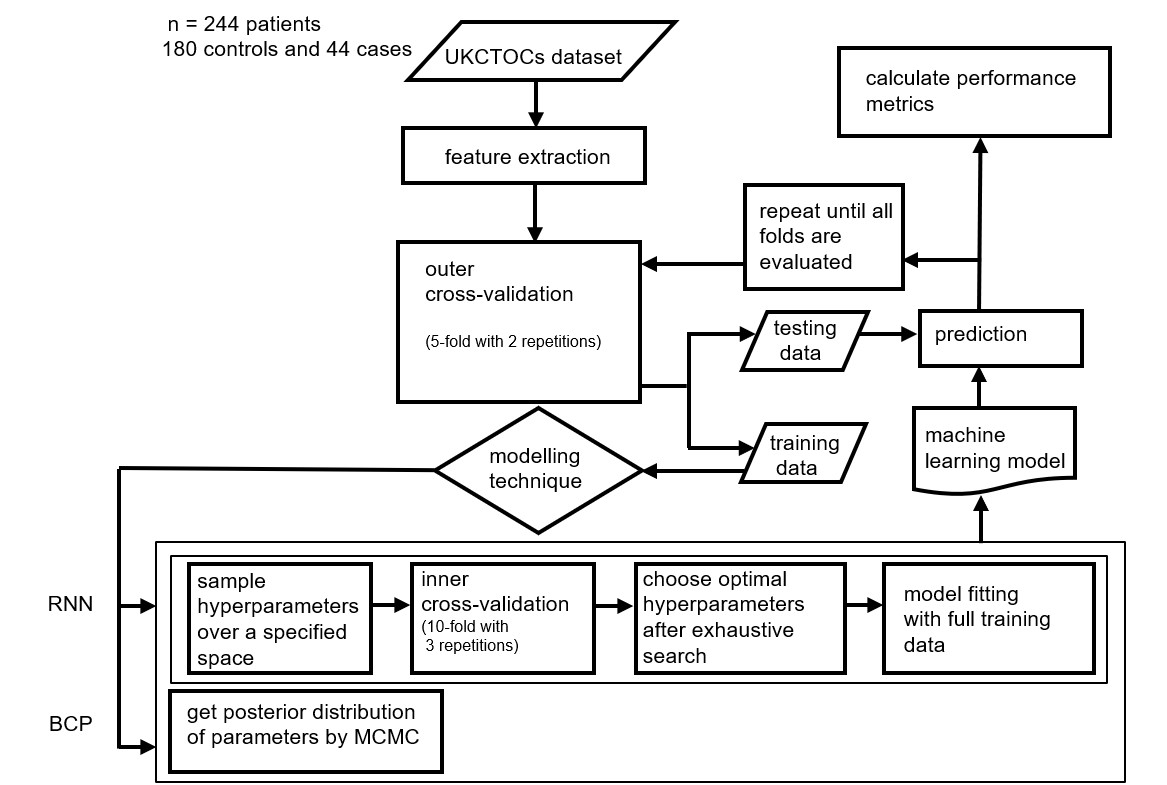


**Supplementary figure 3**: Flow chart describing the design of the study.
